# The burden of household out-of-pocket healthcare expenditures in Ethiopia: a systematic review and meta-analysis

**DOI:** 10.1186/s12939-021-01610-3

**Published:** 2022-01-31

**Authors:** Moges Tadesse Borde, Robel Hussen Kabthymer, Mohammed Feyisso Shaka, Semagn Mekonnen Abate

**Affiliations:** 1 School of Public Health, College of Health Sciences and Medicine, Dilla University, Dilla, Ethiopia; 2 Hawassa, Ethiopia; 3 Department of Nutrition, College of Health Sciences and Medicine, Dilla University, Dilla, Ethiopia; 4 Department of Reproductive Health, College of Health Sciences and Medicine, Dilla University, Dilla, Ethiopia; 5 Department of Anaesthesiology, College of Health Sciences and Medicine, Dilla University, Dilla, Ethiopia

**Keywords:** Household, Out-of-pocket, Healthcare, Expenditures, Ethiopia, Meta-analysis

## Abstract

**Background:**

In Ethiopia, household Out-Of-Pocket healthcare expenditure accounts for one-third of total healthcare expenditure, is one of the highest in the world, and still creates barriers and difficulties for households to healthcare access and may delay or forgo needed healthcare use. Despite the presence of a few highly dispersed and inconsistent studies, no comprehensive study was conducted. Therefore, in this systematic review and meta-analysis, we aimed at estimating the pooled estimates of the burden of household Out-Of-Pocket healthcare expenditures among Ethiopian households and identifying its determinants.

**Methods:**

We systematically searched articles from PubMed / Medline and Google scholar databases and direct Google search engine without restriction on publication period. Cross-sectional and cohort articles and grey literature published in English were included. Data were extracted using Microsoft Excel. Two reviewers screened the titles, reviewed the articles for inclusion, extracted the data, and conducted a quality assessment. The third reviewer commented on the review. Articles with no abstracts or full texts, editorials, and qualitative in design were excluded. To assess quality, Joanna Briggs Critical Appraisal Tools was used. A Forest plot was used to present summary information on each article and pooled common effects. Potential heterogeneity was checked using Cochrane’s Q test and I-squared statistic. We checked publication bias using a Funnel plot. Moreover, subgroup and sensitivity analyses were performed. Meta-analysis was used for the pooled estimates using RevMan statistical software *Version 5.4.1*.

**Results:**

In this review, a total of 27 primary articles were included (with a total sample size of 331,537 participants). Because of the presence of heterogeneity, we employed a random-effects model; therefore, the pooled burden household Out-Of-Pocket / catastrophic healthcare expenditure in Ethiopia was strongly positively associated with household economic status. The odds of facing Out-Of-Pocket / catastrophic healthcare expenditures among the poorest quintile was about three times that of the richest (AOR = 3.09, 95% CI: 1.63, 5.86) *p*-value < 0.001. In addition, on pooled analysis, the mean direct Out-Of-Pocket healthcare expenditures were $32 per month (95%CI: $11, $52) (SD = $45), and the mean indirect Out-of-Pocket healthcare expenditures were $15 per month (95%CI: $3, $28) (SD = $17). The mean catastrophic healthcare expenditure at 10% of threshold was also disproportionately higher: 40% (95%CI: 28, 52%) (SD = 20%). Moreover, the common coping mechanisms were a sale of household assets, support from family, or loan: 40% (95%CI: 28, 52%) (SD = 20%).

**Conclusion:**

Our study revealed the evidence of inequity in financial hardship that the burden of household Out-Of-Pocket / catastrophic healthcare expenditures gap persists among Ethiopian households that is unfair and unjust. To reduce the detected disparities in seeking healthcare among Ethiopian households, national healthcare priorities should target poor households. This calls for the Ministry of Health to improve the challenges and their impact on equity and design better prepayment policies and strengthen financial protection strategies to protect more vulnerable Ethiopian households.

**Protocol registration:**

The details of this protocol have been registered on the PROSPERO database with reference number ID: CRD42021255977.

**Supplementary Information:**

The online version contains supplementary material available at 10.1186/s12939-021-01610-3.

## Background

Every citizen has a natural right to get necessary healthcare in all the stages of life, and must not be hampered by household socioeconomic status [1]. In recent years, policymakers are concerned about the rising Out-Of-Pocket (OOP) healthcare expenditures and their influence on the household economy [2]. This makes health economic evaluation is more important in the healthcare pricing and reimbursement decision-making process.

Household Out-Of-Pocket healthcare expenditures and lack of alternative payment systems, such as insurances are features of the healthcare financing system in low-middle income countries (LMIC) [3]. Many people in low-middle-income countries depend on household Out-Of-Pocket healthcare expenditures any time they receive healthcare services, and unfortunately, they are not protected from financial hardships [4]. Hence, household Out-Of-Pocket healthcare expenditure at a service point is considered an inequitable financing method in the healthcare system [5].

Globally, annually, about 150 million people from 44 million households are impacted by the economic burden of household Out-Of-Pocket healthcare expenditures and nearly more than 100 million from 25 million households are placed into poverty through household Out-Of-Pocket healthcare expenditures [6]. The utilization of healthcare services is impacted by household Out-Of-Pocket healthcare expenditures particularly, people in LMIC, in which on average, household Out-Of-Pocket healthcare expenditures comprise about 40% of healthcare expenditure [7]. Furthermore, in LMIC average household Out-Of-Pocket healthcare expenditure per individual has risen by 66% between 2000 and 2017 [8]. The increased Out-Of-Pocket, particularly in developing countries, can cause economic difficulty, contribute to impoverishment, force households to cut their basic necessities, income loss, and force the household to sell their assets to finance needs for healthcare services [9]. Moreover, poor households may not even be able to afford to seek essential healthcare and they remain trapped in a vicious circle of illness and poverty [9, 10].

In Ethiopia, the burden of household Out-Of-Pocket healthcare expenditures is a significant public health problem and accounts for about 34% (one-third) of total healthcare expenditure, and is one of the highest in the world and is associated with a lack of health insurance coverage and inadequate coverage [11]. Moreover, household Out-Of-Pocket healthcare expenditure is an obstacle for households in accessing healthcare services, however, it is one of the indices to evaluate and address the level of financial risk protection in healthcare systems, and of concern to policymakers and is a key priority in the global health policy agenda and is an indicator of progress toward the Sustainable Development Goals (SDGs) for universal health coverage [11].

In this systemic review and meta-analysis, we developed and applied a conceptual framework that captures elements of our general objective to provide data that could support improvements in healthcare-seeking and reduce financial hardship during healthcare-seeking [12]. Therefore, this study centred on core conceptual domains on identifying problem areas or outcome variables (i.e., the burden of household Out-Of-Pocket healthcare expenditures). The resulting conceptual framework outlines the interconnected factors with our outcome variable such as socioeconomic and demographic factors: (i.e., age, income/household total expenditure, place of residence, educational status, occupation, marital status, and family size); environmental factors (i.e., travel time or distance to health facility and type of health facility visited); having a chronic illness or any illness, and having health insurance.

In Ethiopia, studies indicated that the burden of household Out-Of-Pocket healthcare expenditures was high and has shown a significant increment, indicating 2% of households are facing financial hardship, and this per cent would likely increase with greater health services utilization [13]. In recent years, 46% of rural households in Ethiopia, that sought healthcare faced catastrophic healthcare expenditure at the threshold of 10% of total household expenditure, or 74% at a 40% non-food expenditure, and 92% of households that sought healthcare were pushed further into extreme poverty due to household out-of-pocket healthcare expenditures [14]. Yet, in Ethiopia, Out-Of-Pocket healthcare expenditure could be an obstacle and still create barriers and difficulties for households to healthcare access and may delay or forgo needed healthcare use.

Moreover, to the best of our knowledge, no systematic review and meta-analysis have been carried out to report a consolidated burden of household Out-Of-Pocket healthcare expenditures in Ethiopia. Yet, at the national level, study findings are highly dispersed and inconsistent. We used almost all national representative available data. Evidence on the association between socio-demographic factors, illness status, and household Out-Of-Pocket healthcare expenditures highlights vulnerable households who should be the priority in financial protection policy in Ethiopia.

Furthermore, the burden of household Out-Of-Pocket healthcare expenditures seemed to be high and large numbers of households are suffering from financial hardship in Ethiopia [13]. In this context and within the current literature, this study advances research by generating novel national evidence (pooled estimates) on the burden of household Out-Of-Pocket healthcare expenditures and its influencing factors in Ethiopia. The findings of this study could be input and would help as a base for evidence-based policy intervention and policy practice, and to formulate recommendations that could potentially promote citizen-based healthcare service utilization audit and strengthen community-based health insurance in Ethiopia [15]. In addition, our study is important because it assesses the burden of household Out-Of-Pocket healthcare expenditures, which is one of the monitoring indicators for healthcare financing schemes in the country. The study would also be of paramount importance for researchers to do in related topics. Therefore, in this systemic review and meta-analysis, the purpose was to provide a comprehensive understanding of the burden of financial hardship for healthcare-seeking in Ethiopia and to advance research by generating novel national evidence (pooled estimates) household Out-Of-Pocket healthcare expenditures and their influencing factors with the aim to assess the burden of household Out-Of-Pocket healthcare expenditures in Ethiopia. The review question was: what was the extent of the burden of Out-Of-Pocket healthcare expenditures that were associated with poor socioeconomic status, compared to the rich, in Ethiopian households with illnesses?

## Methods

### Reporting and registration of the protocol

The report of this systematic review and meta-analysis was written using an updated PRISMA 2020 guideline (i.e, Preferred Reporting Items for Systematic Review and Meta-Analysis) [16]. The details of this systematic review and meta-analysis have been registered on the PROSPERO database (i.e., International Prospective Register of Systematic Reviews) with reference number ID: CRD42021255977 [17]. The completed checklist has been provided as supplementary material (Additional file 1).

### Eligibility criteria

We reviewed articles using defined inclusion and exclusion criteria. Briefly, it would be articles that contain information answering our research question. But, the most important is that it should be clear and sufficient information, including positive or negative, to answer the question.

#### Inclusion criteria

##### **Study type**

All observational articles (cross-sectional, and cohort or longitudinal) and grey literature reporting the target outcomes, i.e., household Out-Of-Pocket healthcare expenditures and their determinants in Ethiopia were included.

##### **Setting**

Both community and institutional-based articles conducted in Ethiopia were included.

##### **Language**

Articles written and published in the English language were included.

##### **Population**

The participants were Ethiopian households or individuals with and without experience of Out-Of-Pocket healthcare expenditures.

##### **Publication status**

Articles available as full-text, peer-reviewed, and published articles were included.

##### Publication status

All published articles.

##### Publication year

There was no restriction on the publication period.

##### Publication date

We included articles published until 31 June 2021.

#### Exclusion criteria

Articles that were unrelated or duplicated or overlapping data, articles that didn’t report full texts or were unavailable in full texts, articles with data not reliably extracted, articles that didn’t report household Out-Of-Pocket healthcare expenditures, articles with different outcomes of interest, studies with a methodological score less than 50%, with randomized controlled trials, systematic review, case report, case series, editorials, author response, theses, and books, articles that had no abstracts or abstract-only papers as preceding papers, conference, editorial, and commentaries, articles conducted in nonhuman subjects, articles that qualitative in design, and those not published in the English language were excluded.

### Information sources

For this systematic review and meta-analysis, a primary literature search was carried out using PubMed/Medline and Google scholar electronic databases and a manual direct Google search engine from June 1 to 31, 2021.

### Search strategy

To ensure consistency across articles and to ensure comprehensiveness, a common and comprehensive four-stage systematic review of primary studies was employed. An initial search was carried out on PubMed/Medline and Google scholar electronic databases, followed by an analysis of the text words contained in the title/abstract and indexed terms. A second search combined free text words and indexed terms with Boolean operators and key terms: (prevalence OR incidence OR burden) AND household Out-Of-Pocket healthcare expenditures OR Out-Of-Pocket healthcare expenditures OR catastrophic healthcare expenditures OR impoverishing healthcare expenditures AND Ethiopia. In other words, (financial risk OR financial catastrophe OR health equity) AND (factors OR risk factor OR risk OR determinant) AND household Out-Of-Pocket healthcare expenditures AND Ethiopia. The third search used reference lists of all identified eligible articles to identify other additional pertinent articles to this review. Finally, an additional search was conducted on the Google search engine manually. The primary search strategy was largely limited to English language publications as deemed appropriate based on a review of the primary and secondary search results. Articles retrieved from various electronic databases and Google search engines were exported to EndNote reference manager software version X7 (Thomson Reuters, Stamford, CT, USA) where duplicate articles could be identified and removed. Some duplicates were also addressed manually due to variations in reference styles across sources. We kept a log of all reviewed articles.

### Study records

#### Data management

We adhered to an updated PRISMA 2020 guideline [16] to assess the identified articles by their titles, abstracts, as well as full-text contents against the predefined eligibility criteria.

#### Selection process

During the selection process, the selection of articles was carried out by two independent reviewers in two phases: the first phase was that for the eligibility of the studies, unnecessary data was excluded by reading, screening, or reviewing the titles and abstracts of each article yielded by the comprehensive search. The second phase was that after reading the abstracts of the articles that were potentially relevant to this review, we reviewed the full texts against the selection criteria. During this article selection process, articles; which were not fully accessed, unclear, vague, incomplete, or any relevant information was missing from the reviewed articles; were excluded. However, before excluding the articles, the corresponding author of the study was contacted via e-mail at least two attempts to retrieve the missing information every 2 weeks apart.

#### Data collection process /data extraction or abstraction/

Relevant data from each study was extracted in duplicate by two independent reviewers from source documents using a customized, standardized, beforehand piloted data collection form in Microsoft Excel. After screening, all full-text articles were assessed for eligibility and were included in a summary table (Additional file 2). Subsequently, both reviewers examined the extracted data and determine the level of evidence. Any differences in opinion between the two independent reviewers were resolved consensus-based discussion. If insufficient, this was followed by a referral to a third reviewer to fix the matter at hand.

As it is recommended by PRISMA guideline [18], pre-specified data elements were extracted from each article for primary outcome: including quantitative findings such as the number of participants (households / individuals) who experienced Out-Of-Pocket healthcare expenditures (‘Yes’), and those who did not experience Out-Of-Pocket healthcare expenditures (‘No’), total households/individuals (n), and AOR (95% CI) were presented on the column of the data collection form. The primary outcome also included qualitative information: like the name of the first author (principal author), publication year, title and journal, study area (country and region), study design, year of study, setting/location, sample size, response rate, and the tool also presented on the data collection form. In the meantime, for the secondary outcome (predictors), the data included socio-demographic variables (gender mean of age of the participants, and socioeconomic status of households/individuals) if at least two or more studies reported them as a predictor. We selected these variables because they are the most frequently reported predictors by the sample studies identified for this meta-analysis. For each predictor, to calculate the odds ratio, we extracted the data from the primary studies in the form of two by two tables. Any deviations from the central predetermined inclusion and exclusion criteria were noted and justified. Whenever variations of extracted data were observed, all the steps were repeated.

#### Data items /definition of variables/

For the aim of this study, a systematic review was defined as a *review* of the available evidence on a formulated question using an explicit plan and systematic search strategy to identify, select, critically appraise, and combine relevant and reliable primary research, and to extract and analyze data from the articles that were included in the *review* [19]. While meta-analysis was *defined* as a statistical method used to pool or systematically combine, summarize, and interpret the results from more than one previous study to derive conclusions about that body of research [20]. Household total expenditure is defined as the sum of household food consumption expenditure and non-food consumption expenditures of the household. Household Out-Of-Pocket expenditures covered expenditures made by a patient or a household for care and treatment.

#### Outcome measurement

This systematic review and meta-analysis had two main outcomes.

#### Primary outcomes

The primary outcomes were the burden of household Out-Of-Pocket healthcare expenditures in Ethiopia. The burden of household Out-Of-Pocket healthcare expenditures was measured using either its share of total household income or its share of total household consumption. Therefore, household Out-Of-Pocket expenditures was that made by a patient or a household for care and treatment; for example, included payments for card fees or consultation fees, medication, laboratory tests, and health facility or hospital bills where insurance does not cover the full cost of the healthcare services [21]. Household payment for utilisation of healthcare services was the summation of the household’s *direct medical* and *direct non-medical* Out-Of-Pocket healthcare expenditures. A household’s *direct medical* Out-Of-Pocket healthcare expenditures were calculated in terms of direct payment made by households to healthcare providers at the point of receiving healthcare services. This included registration/card fees, medicines, laboratory tests, etc., for outpatient visits; and for inpatient stays, bed charges at healthcare facilities.

Direct costs are costs associated with card fees /consultation, diagnostic workup medications, and transportation. Household’s *direct medical* Out-Of-Pocket healthcare expenditures have excluded any prepayment for healthcare services, i.e. taxes or insurance.

Household’s *direct non-medical* Out-Of-Pocket healthcare expenditures were calculated in terms of payments related to transportation, and daily living payments including accommodation, and food for the accompanying household members or caregivers, and additional expenses for the caregiver during outpatient and inpatient visits [22]. Thus, the burden of household Out-Of-Pocket healthcare expenditure was calculated as its share of total household income or its share of total household consumption [21] of households/participants who have been included in the study (sample size) multiplied by 100.

Catastrophic health expenditure, households whose financial contributions to the health care exceeded 10% of total household expenditure or 40% of non-food household expenditure or income were considered as exposed to catastrophic healthcare expenditures.

#### Secondary outcomes

The risk factors of the burden of household Out-Of-Pocket healthcare expenditures in different regions of the country were secondary outcomes, such as socioeconomic and demographic factors (i.e., age, income/household total expenditure, place of residence, educational status, occupation, marital status, and family size); environmental factors (i.e., travel time or distance to health facility and type of health facility visited; and health insurance); and having a chronic illness or any illness.

#### Risk of bias in individual studies /quality assessment/

To evaluate the quality of the articles, the trustworthiness and relevance of the results of published articles (reporting, external and internal validity) were evaluated using the Joanna Briggs Institute’s standardized critical appraisal tools [23] (Additional file 3). Articles with an average of greater than 50% (four out of eight) scores for cross-sectional studies and 50% (six out of twelve) scores for cohort studies in Joanna Briggs Institute’s standardized critical appraisal were considered as low risk and included in the study. Moreover, the quality of those articles that passed the Joanna Briggs Institute’s critical appraisal, was also further evaluated using a modified version of ‘The Newcastle-Ottawa Scale (NOS)’ [24] (Additional file 4). ‘The Newcastle-Ottawa Scale (NOS)’ has three major domains and is graded out of 10 points (stars): 1) the first section assesses the methodological quality of each study and weighs a maximum of five stars; 2) the second section considers the comparability of the study and takes two stars; 3) the last part of the tool assesses outcomes with related to statistical analysis.

To assess the trustworthiness, relevance, and quality of the articles, the following items were used to appraise, for example, for case-control studies: (1) comparable groups, (2) cases and controls matched, (3) the same criteria used for identification, (4) exposure measured in a standard, valid and reliable way, (5) exposure measured in the same way for cases and controls, (6) confounding factors identified, (7) strategies to deal with confounding factors, (8) outcomes assessed in a standard, valid and reliable way for cases and controls, (9), the exposure period of interest long enough, and (10) appropriate statistical analysis [25]. Cohort articles were also appraised by using the following items: (1) the two groups similar and recruited from the same population, (2) the exposures measured similarly to assign people to both exposed and unexposed groups, (3) the exposure measured using valid and reliable methods, (4) confounding factors identified, (5) strategies to deal with confounding factors, (6) participants free of the outcome at the start of the study, (7) the outcomes measured using valid and reliable methods, (8) follow up time reported and sufficient to be long enough for outcomes to occur, (9) follow up complete, and if not, were the loss reasons to follow up described and explored, (10) strategies to address incomplete follow up, and (11) appropriate statistical analysis [25]. For cross-sectional articles the following items were used to appraise the quality: (1) criteria for inclusion, (2) study subjects and the setting described, (3) exposure measured using valid and reliable methods, (4) standard criteria used for measurement, (5) confounding factors identified, (6) strategies to appropriate statistical analysis deal with confounding factors, (7) outcomes measured using valid and reliable methods, and (8) appropriate statistical analysis [25].

To assess the trustworthiness, relevance, and quality of economic evaluation studies, the following items were also used to appraise: (1) the question defined, (2) alternatives described, (3) costs and outcomes identified, (4) clinical effectiveness established, (5) costs and outcomes measured accurately, (6) costs and outcomes valued credibility, (7) costs and outcomes adjusted for differential timing, (8) incremental analysis of cost and consequences conducted, (9), sensitivity analysis conducted, (10) issues of concern to users included, and (11) generalizability of findings in the review [26].

#### Data synthesis and analysis

Selected articles were rated for level of evidence and methodological quality, information that was also included. Based on the evidence compiled, answers to the targeted questions were formulated and anonymized voting was done. A level of consensus of 80% or higher was considered to represent a strong agreement. Subsequently, study data were quantitatively synthesized.

The necessary data were extracted using a Microsoft Excel format, and then it was imported and the Meta-analysis was done by using RevMan software *Version 5.4.1* [27]*.* Data were tabulated, and a narrative synthesis was presented. A descriptive statistical analysis was performed to summarize the extracted data. The standard error of prevalence/incidence for each original article was calculated by the binomial distribution formula.

Since the included articles may exhibit high heterogeneity, a random effect meta-analysis model was computed to determine the pooled estimate of the burden of household Out-Of-Pocket healthcare expenditures [28]. A Forest plot was used to summarize information on each study and show the estimated common effect, and all in one figure. The associated factors were qualitatively narrated or synthesized accordingly (socioeconomic and demographic factors, environmental factors, behavioural factors, and illness status). Moreover, the association between predictor variables, and household Out-Of-Pocket healthcare expenditures were examined.

The Cochrane’s Q and I^2^ statistic was used to check heterogeneity among included articles and to identify which articles contribute to the observed heterogeneity as part of outlier and influence analyses, where significant heterogeneity was assumed if the *P*-value for the Q test < 0.10 and I^2^ value > 50% (the values of 25, 50, and 75% represented as low, moderate, and high level of heterogeneity, respectively) [29]. However, if articles were heterogeneous (I^2^ > 50%), the results of each study were narrated. However, in this study, an I^2^ value of less than 50% was considered to interpret the combined effect size.

Subgroup analyses (also known as moderator analyses) were carried out based on the region of studies and sample size, to assess why some studies have higher or lower true effect sizes than others or find excess variability (patterns of heterogeneity) in studies and what causes it**.**

Sensitivity analysis was also conducted to assess the effects of a single study on determinants of the burden of household Out-Of-Pocket healthcare expenditure [30].. For the small number of articles, it may be impossible to estimate the between studies variance with any precision. Therefore, we used a fixed-effect model for less than five articles and a random effect model for five and above articles [31]. Moreover, univariate meta-regression was conducted by taking the publication year to explain the true effect size differences in the studies (the between-study heterogeneity variance), and the sample size of the studies to identify the possible source of heterogeneity.

#### Meta-bias (es)

Meta-bias, presence of potential publication bias, and selective reporting within articles were assessed subjectively by using a Funnel plot through a visual inspection of the symmetry of the amount of study heterogeneity [32].

#### Confidence in cumulative evidence

The level of significance was set at 95% confidence level and *P* < 0.05.

## Results

### Search results

**Fig. 1:**
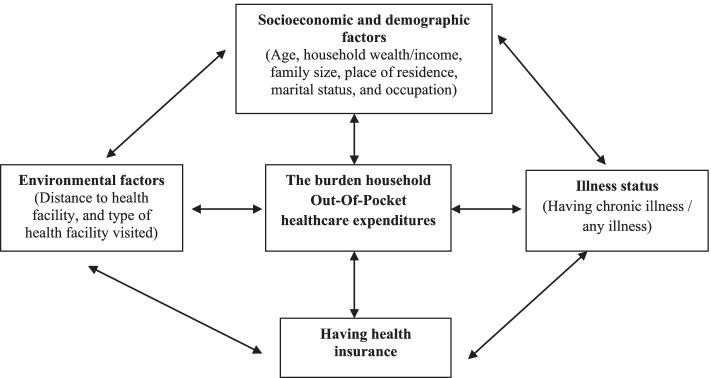
The conceptual framework of determinants the burden of household Out-Of-Pocket healthcare expenditure in Ethiopia, 2021

The search strategy retrieved 210 articles from databases and other sources. Of these initial records, 98 articles were excluded due to duplication. From the remaining 112 articles, 80 articles were excluded for reasons as their titles and abstracts did not match (= 62) and some articles were irrelevant to study questions (= 18). Therefore, 32 full-text articles were accessed, and assessed for eligibility based on the pre-set criteria, which resulted in the further exclusion of five articles primarily due to some articles were not being published in peer-reviewed journals (= three) (i.e., thesis) and some did not report outcome interest (= two). Then, 27 articles with a sample size of 331,537 participants were included to assess determinants of the burden of household Out-Of-Pocket healthcare expenditures. Of these, six articles were conducted at the national level [13, 33–37]. The remaining 21 articles were conducted in different parts of Ethiopia: i.e., six articles were conducted in the south regional state [14, 33–37], five articles in Amhara regional state [38–42], five articles in Addis Ababa city [43–47], three articles in Oromiya regional state [48–50], one article in Bensangual-Gumuz regional state [51], and one article in a combination of Afar and Oromiya regional states [52]. Finally, 17 articles were found to be eligible and included in the meta-analysis (Fig. 1).

### Characteristics of included articles

Twenty-one articles were done by cross-sectional study design [33, 35–54], two articles by longitudinal cohort study design [14, 34], one article by a combination of retrospective and retrospective cohort study design [55], one report of national data [56], and two articles that used secondary data from a survey [13, 57] (Table 1). Moreover, these studies were conducted from 2005 and 2021.

**Table 1:** Descriptive summary of 27 articles reporting the burden household Out-Of-Pocket healthcare expenditures in Ethiopia, 2021

Author (year) (reference number)	Region/ study area	Study design	Sample size (N)	Quality score	Quality status
World Bank (2005) [56]	National	Report	Not specified	6	Low risk
Tadele B. et al. (2005) [48]	Oromiya	Cross-sectional	630 households	7	Low risk
Wakgari D. et al. (2007) [49]	Oromiya	Cross-sectional	2195 households	6	Low risk
Pearson L et al. (2011) [53]	National	Assessment/ Cross-sectional	751 health facilities (112 hospitals and 639 health centres).	6	Low risk
Tibebe Akalu, et al. (2012) [33]	South	Cross-sectional	1015 households	5	Low risk
Mekuanenet G. et al. (2015) [38]	Amhara	Cross-sectional	467 employees	7	Low risk
Getahun B. et.al. (2016) [43]	Addis Ababa	Cross-sectional	604 patients	7	Low risk
Tolla MT. et.al. (2017) [44]	Addis Ababa	Cross sectional	625 patients	6	Low risk
Memirie ST. et.al. (2017) [55]	National	Retrospective and Prospective cohort	686 patients	6	Low risk
Bedane SN et.al. (2018) [45]	Addis Ababa	Cross-sectional	422 patients	7	Low risk
Mekonen AM. et.al (2018) [39]	Amhara	Cross-sectional	454 households	5	Low risk
Fitsum S. et al. (2018) [40]	Amhara	Cross-sectional	346 patients	8	Low risk
Abyot A. et al. (2018) [34]	South	Longitudinal	735 patients	7	Low risk
Hailemichael Y. et.al (2019) [35]	South	Comparative cross-sectional	579 households	7	Low risk
Hailemichael Y. et.al. (2019) [36]	South	Comparative ross-sectional	257 households	7	Low risk
Biniam G. et al. (2019) [37]	South	Cross-sectional	812 women	7	Low risk
Amelewerk A. et al. (2019) [46]	Addis Ababa	Cross-sectional	422 patients	7	Low risk
Miljeteig I. et al. (2019) [54]	National	Survey/ Cross-sectional	647 physicians	8	Low risk
Obse AG. et.al. (2020) [57]	National	Data from the Ethiopian Household Consumption Expenditure Survey (HCES) 2010/11	10,368 rural and 17,664 urban households	7	Low risk
Debelo S. et.al. (2020) [51]	Benishangul-Gumuz	Cross-sectional	488 households	7	Low risk
Mizan K. et al. (2020) [13]	National	Data from the 2015/16 Ethiopian household consumption and expenditure and welfare monitoring surveys	30, 229 households	6	Low risk
Addisu B. et al. (2020) [50]	Oromiya	Cross-sectional	354 patients	7	Low risk
Assebe LF. et.al. (2020) [52]	Afar and Oromiya	Cross sectional	1006 HIV and 787 TB participants	5	Low risk
Borde MT. et.al. (2020) [14]	South	Prospective cohort	2350 participants	7	Low risk
Kasahun GG. et.al. (2020) [47]	Addis Ababa	Cross-sectional	404 participants	6	Low risk
Tsega G. et al. (2021) [41]	Amhara	Cross-sectional	422 participants	7	Low risk
Yohannes S. et al. (2021) [42]	Amhara	Cross-sectional	302 chronically ill patients	6	Low risk

### Risk of bias within articles

**Fig. 2:**
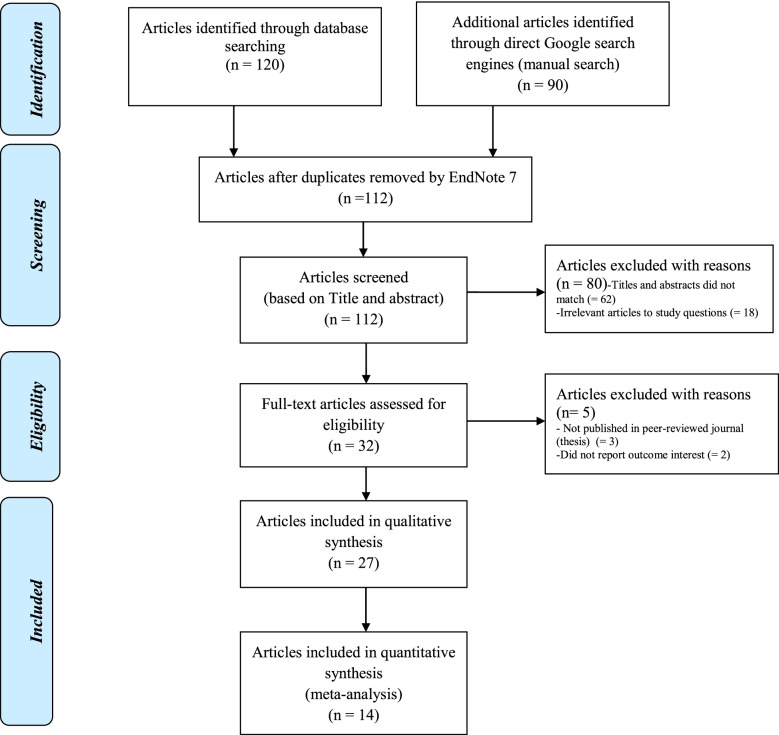
PRISMA study flow diagram [71] describing the selection of articles for the systematic review and meta-analysis of the burden of household Out-Of-Pocket healthcare expenditures in Ethiopia, 2021 (showing data collection process, identified, screened, eligible, and included studies). Articles may have been excluded for more than one reason

By using Joanna Briggs Critical Appraisal Tools for review and meta-analysis for cross-sectional articles and cohort articles, those studies that had low risk were included for the review (Table 1, Fig. 2).

### The burden of household out-of-pocket healthcare expenditures

#### Direct out-of-pocket healthcare expenditures

The pooled analysis of 21 studies identified that the mean direct Out-of-Pocket healthcare expenditures during utilization of healthcare services was $32 per month in Ethiopia (95%CI: $11.5, $52.5) (SD = $45.1) [14, 33, 34, 36, 37, 39–41, 43, 45–53, 55–57].

#### Indirect out-of-pocket healthcare expenditures

The pooled analysis of 10 studies identified that the mean indirect Out-of-Pocket healthcare expenditures during utilization of healthcare services were $15.6 per month in Ethiopia (95%CI: $3.0, $28.2) (SD = $17.6) [33, 41, 43, 45, 47, 49, 52, 55].

### Catastrophic healthcare expenditure

The pooled analysis of 14 studies identified that the mean catastrophic healthcare expenditures at 10% of threshold during utilization of healthcare services was 40.1% in Ethiopia (95%CI: 28.7, 52.2%) (SD = 20.4%) [13, 14, 35, 36, 38–42, 44, 47, 48, 50–52, 56].

### Coping mechanisms

The pooled analysis of 11 studies identified that the common coping mechanisms during utilization of healthcare services in Ethiopia were a sale of household assets, support from family and relatives, and loan: 40.1% (95%CI: 28.7, 52.2%) (SD = 20.4%) [14, 33, 36, 38, 41, 43, 44, 47–49, 52].

### Meta-analysis

As shown in the forest plot, a quantitative pooled analysis of 14 included articles revealed that the pooled burden household Out-Of-Pocket healthcare expenditures in Ethiopia were strongly positively associated with household economic status. The odds of facing Out-Of-Pocket / catastrophic healthcare expenditures among the poorest quintile was about three times that of the richest (AOR = 3.09, 95% CI: 1.63, 5.86) *p*-value < 0.001, (Table 2). Nonetheless, extreme heterogeneity was exhibited across the studies and uncovered by *P*-value for Cochrane’s Q test (*p*-value *<* 0.001) and I^2^ statistic (I^2^ = 95%). As a result, a random effect model was employed to estimate the pooled burden of household Out-Of-Pocket healthcare expenditures in Ethiopia.

**Table 2:**
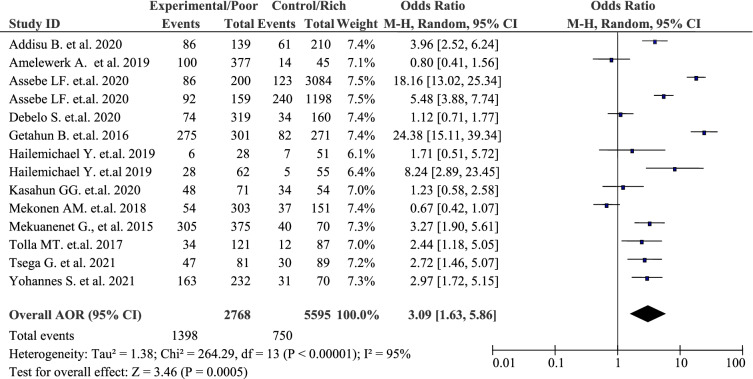
The forest plot of the pooled estimates of the burden household Out-Of-Pocket healthcare expenditures in Ethiopia, 2021

### Factors associated with the burden of household out-of-pocket healthcare expenditures

In this study, socioeconomic and demographic factors; environmental factors; illness status; and healthcare-seeking behaviour (i.e., insured households) were independent predictors of the burden of household Out-Of-Pocket healthcare expenditures in Ethiopia.

The factors such as socioeconomic and demographic factors (i.e., age, household wealth/income, family size, place of residence, marital status, and occupation); environmental factors (i.e., distance to health facility, and type of health facility visited); and having health insurance, exhibited that *P*-value for the Cochrane’s Q test < 0.10 and I^2^ statistic > 50% and this indicated that the included articles were heterogeneous. However, the result of each factor was narrated.

However, for factors such as socioeconomic and demographic factors (i.e., educational status) and illness status (i.e., having a chronic illness / any illness) exhibited that the Cochrane’s Q test > 0.10 and I^2^ statistic < 50% and this indicated that the included articles were not heterogeneous; therefore, the combined effect size of these two factors was further interpreted.

### Socioeconomic and demographic factors

The pooled analysis of four studies identified that age increased the probability of Out-Of-Pocket / catastrophic healthcare expenditure (AOR 1.02, 95% CI: 0.98, 1.05) [36, 42, 44, 47] (Table 3). Age < 30 years (AOR 7.74, 95% CI: 0.94, 63.62; *P* = 0.01) was associated with catastrophic healthcare expenditure [42]. In other words, increasing catastrophic healthcare expenditure with age could be attributed to illness complications [47].

**Table 3:**
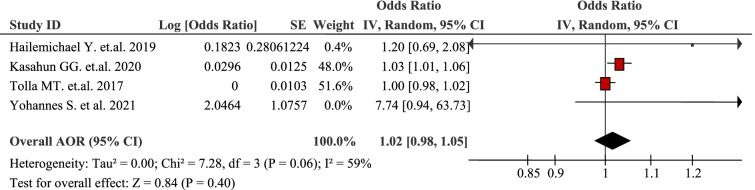
Age as a factor in four articles reporting the burden household Out-Of-Pocket healthcare expenditures in Ethiopia, 2021

The pooled analysis of seven studies disclosed that household wealth / income was positively associated with the burden of household Out-Of-Pocket / catastrophic healthcare expenditure (AOR 2.58, 95% CI: 1.31, 5.06) (Table 4). Poor households were 2.4 times (AOR = 2.417; CI: 1.079, 5.413) more likely to encounter catastrophic healthcare expenditure as compared with that of rich households [41]. Income level was strongly negatively associated with catastrophic healthcare expenditure. The odds of facing catastrophic healthcare expenditure among the poor households was about 60 times that of the richest (OR = 58.62, 95% CI:16.2, 208.0) [44].

**Table 4:**
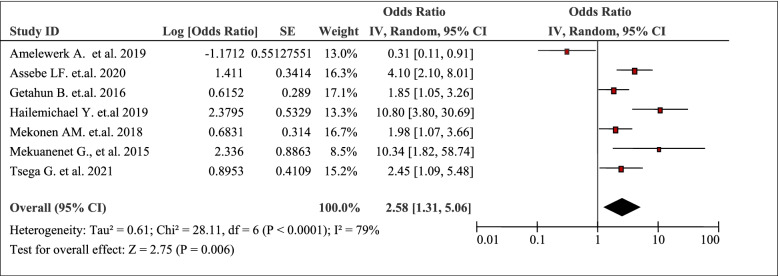
Household wealth / income as a factor in seven articles reporting the burden household Out-Of-Pocket healthcare expenditures in Ethiopia, 2021

The pooled effects of five studies revealed that household size / family size was one of the factors for the burden of household Out-Of-Pocket / catastrophic healthcare expenditures (AOR 0.89, 95% CI: 0.60, 1.32) (Table 5). There was a linear relationship between family size and catastrophic healthcare expenditure [44]. The cost of illness increased with an increase in family size, where the cost was higher in patients with family sizes of 4–6.

**Table 5:**
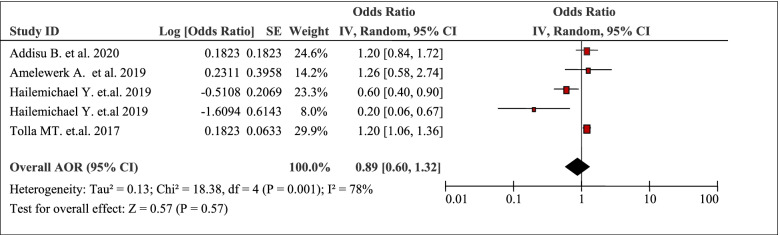
Household size / family size as a factor in five articles reporting the burden household Out-Of-Pocket healthcare expenditures in Ethiopia, 2021

(AOR 1.20, 95% CI: 0.84,1.72) compared to patients with family size of 1–3 [50].

The pooled analysis of five studies displayed that place of residence was positively associated with the burden of household Out-Of-Pocket / catastrophic healthcare expenditures (AOR 3.02, 95% CI: 1.39, 6.57) (Table 6). Dwelling in rural areas, in the case of cardiovascular patients (AOR 3.25; 95%CI: 1.79, 5.90) and the case of tuberculosis patients (AOR 3.15; 95%CI: 1.65, 6.03) was associated with household Out-Of-Pocket or catastrophic healthcare expenditures [43, 44].

**Table 6:**
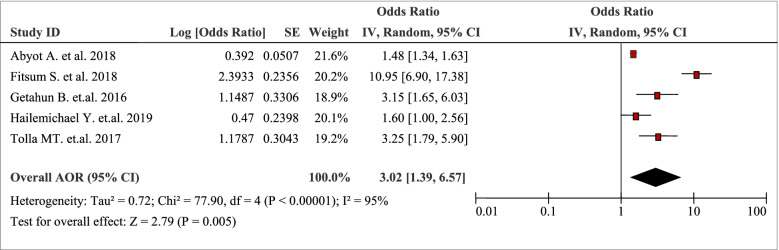
Place of residence as a factor in five articles reporting the burden household Out-Of-Pocket healthcare expenditures in Ethiopia, 2021

The pooled effects of three studies showed that educational status was associated with the burden of household Out-Of-Pocket / catastrophic healthcare expenditures (AOR 0.92, 95% CI: 0.87, 0.97) (Table 7). Patients with no formal education have a higher total cost of hypertension illness (AOR 0.93; 95%CI, 0.88, 0.98) [50] and have a higher pre-and post-diagnosis cost of tuberculosis patients (AOR 0.87; 95%CI, 0.77, 0.97) [34] compared to patients with a primary education level.

**Table 7:**
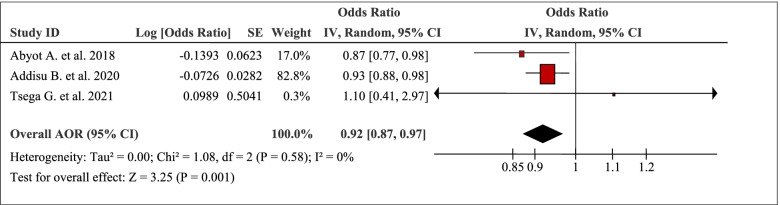
Educational status as a factor in three articles reporting the burden household Out-Of-Pocket healthcare expenditures in Ethiopia, 2021

The pooled analysis of three studies revealed that marital status was associated with the burden of household Out-Of-Pocket / catastrophic healthcare expenditures (AOR 10.25, 95% CI: 0.92, 114.21) (Table 8). Marital status was associated total Out-Of-Pocket healthcare expenditures (AOR 0.20, 95%CI: 0.19, 0.20) [46]. However, being unmarried was found to be associated with higher median total cost (AOR 1.17, 95%CI: 0.02, 55.40) [40, 46].

**Table 8:**
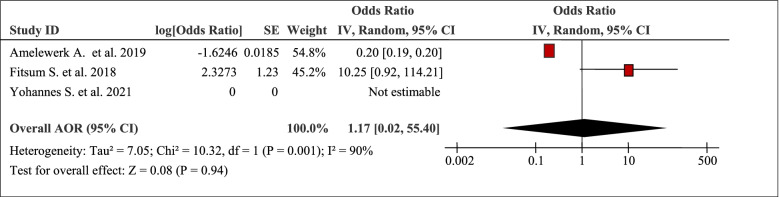
Marital status as a factor in three articles reporting the burden household Out-Of-Pocket healthcare expenditures in Ethiopia, 2021

The pooled effects of three studies showed that occupation was associated with the burden of household Out-Of-Pocket / catastrophic healthcare expenditures (AOR 0.53, 95% CI: 0.28, 1.03) (Table 9). Type of occupation of the patients was associated with total Out-Of-Pocket healthcare expenditures (AOR 0.84, 95%CI: 0.83, 0.85) [46]. For example, households that had a member of working adults were 68% (AOR = 0.32; 95% CI: 0.16, 0.63) times less likely to face catastrophic healthcare expenditures compared to households who had no working adults [51].

**Table 9:**
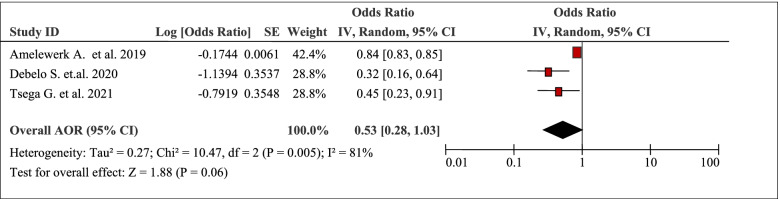
Occupation as a factor in three articles reporting the burden household Out-Of-Pocket healthcare expenditures in Ethiopia, 2021

### Illness status

The pooled effects of seven studies identified that having a chronic illness / any illness was positively associated with the burden of household Out-Of-Pocket / catastrophic healthcare expenditures (AOR 2.23, 95% CI: 1.77, 2.81) (Table 10). The odds of facing catastrophic healthcare expenditures among hospitalized subjects was about eight times that of the non-hospitalized subjects (OR = 8.39, 95% CI: 4.24, 16.59) [44]. Moreover, household members with any chronic illnesses were 3.93 (AOR 3.93, 95%CI: 1.78, 9.14) times more likely to encounter catastrophic healthcare expenditures than household members without chronic illnesses [51].

**Table 10:**
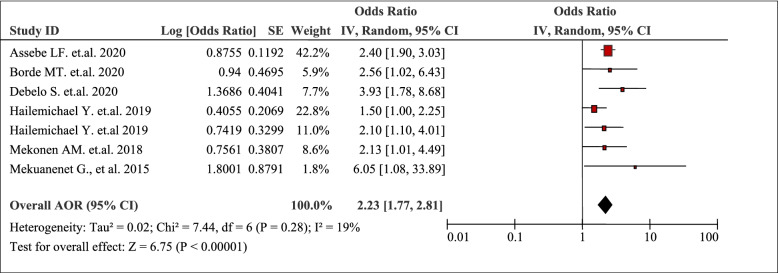
Having chronic illness / any illness as a factor in seven articles reporting the burden household Out-Of-Pocket healthcare expenditures in Ethiopia, 2021

### Environmental factors

The pooled effects of two studies showed that distance to health facilities was associated with the burden of household Out-Of-Pocket / catastrophic healthcare expenditures (AOR 0.06, 95% CI: 0.00, 18.53) (Table 11). Travel time to treatment centre beyond 1 h predicted increased total patient cost of TB care (AOR 1.09, 95% CI: 1.02, 1.16) [34]. Moreover, distance from the hospital (≥10 km), was the predictor of Out-Of-Pocket/catastrophic healthcare expenditures during hypertension [50].

**Table 11:**
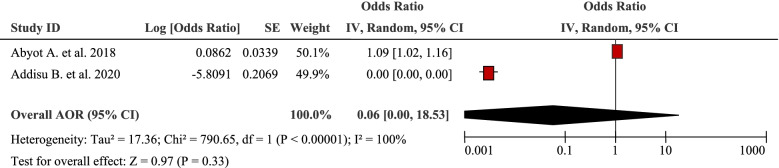
Distance to a health facility as a factor in two articles reporting the burden household Out-Of-Pocket healthcare expenditures in Ethiopia, 2021

The pooled analysis of four studies pointed out that type of health facility visited was positively associated with the burden of household Out-Of-Pocket / catastrophic healthcare expenditures (AOR 4.44, 95% CI: 0.92, 21.40) (Table 12). Seeking healthcare in private hospitals increased the odds of catastrophic healthcare expenditures by 20 fold (OR = 20.7, 95% CI: 10.2, 42.04) compared with public hospitals [44].

**Table 12:**
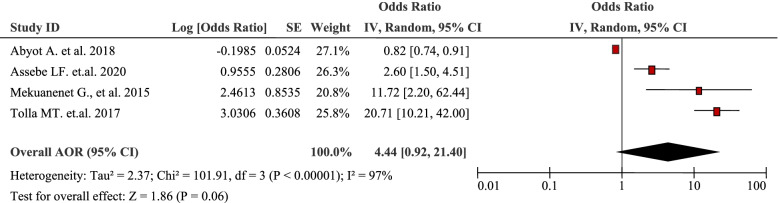
Type of health facility visited as a factor in four articles reporting the burden household Out-Of-Pocket healthcare expenditures in Ethiopia, 2021

### Health seeking behaviour

The pooled effects of two studies illustrated that insured households were less likely to experience household Out-Of-Pocket / catastrophic healthcare expenditures (AOR 0.70, 95% CI: 0.05, 9.41) (Table 13). For example, insured households were 81% times less likely to incur catastrophic health expenditure (AOR = 0.19, 95% CI: 0.11–0.33) compared with non-insured households [39]. Moreover, households with a health insurance scheme have protection from catastrophic healthcare expenditures (AOR 2.7; 95% CI 1.10 to 6.63) [52].

**Table 13:**
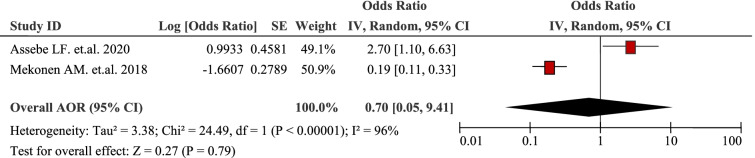
Being insured household as a factor in two articles reporting the burden household Out-Of-Pocket healthcare expenditures in Ethiopia, 2021

### Subgroup analysis

Subgroup analysis was performed to resolve study variations. In this meta-analysis, we performed subgroup analysis based on the year of publication with heterogeneity. Based on the subgroup analysis, the odds of the estimated pooled burden of household Out-Of-Pocket healthcare expenditures in Ethiopia was higher in articles published in the year after 2019 (AOR 3.95, 95%CI 1.70, 9.18) than the year before and in 2019 (AOR 2.55, 95%CI 0.91, 7.15) (Table 14).

**Table 14:** Subgroup analysis of the pooled estimate of the burden of household Out-Of-Pocket healthcare expenditures in Ethiopia, 2021 using AOR, 95%CI, the *p*-value for X^2^, and heterogeneity estimate among Ethiopian households or individuals with OOP / CHE

Subgroup analysis by	Characteristics	Pooled estimate (AOR, 95%CI)	(***p-***value for ***X***^***2***^)	***I***^***2***^
Year of publication	Before and at 2019	2.55 (0.91, 7.15)	*p*-value, 0.001	95%
	After 2019	3.95 (1.70, 9.18)	*p*-value, 0.001	96%
Test for subgroup differences: *X*^*2*^ = 0.41, df = 1, (*p* = 0.52), *I*^*2*^ = 0%				

Here, the *p*-value of the subgroup for the articles published in the year before and in 2019 was not significant (*p*-value 0.07). However, the *p*-value of the subgroup for the articles published in the year after 2019 was significant (*p*-value 0.001). This indicated that the two subgroups were not identical. Yet, a test for subgroup differences was done to see the difference between the two groups. However, the test for subgroup differences was not statistically significant (*p*-value 0.52). This indicated that there was no publication difference in the two subgroups. Moreover, the presence of high heterogeneity was dependent on the publication year (Table 14).

### Sensitivity analyses

Sensitivity analysis was employed to remove inferior quality articles and to identify possible outlying articles and the effects of a single study on the overall estimation. To check the effect of a single study on the overall outcome, we used the leave-one-out method. We excluded each study, in turn, to see if it leads to a remarkable change in the fitted random-effects model. If it happens, then the study may be considered to be influential; if not, then the study may impose little influence on the results. Therefore, in all cases, there was no observed article that exerted a significant impact on the overall estimate of the burden of household Out-Of-Pocket healthcare expenditures in Ethiopia.

### Publication Bias

**Fig. 3:**
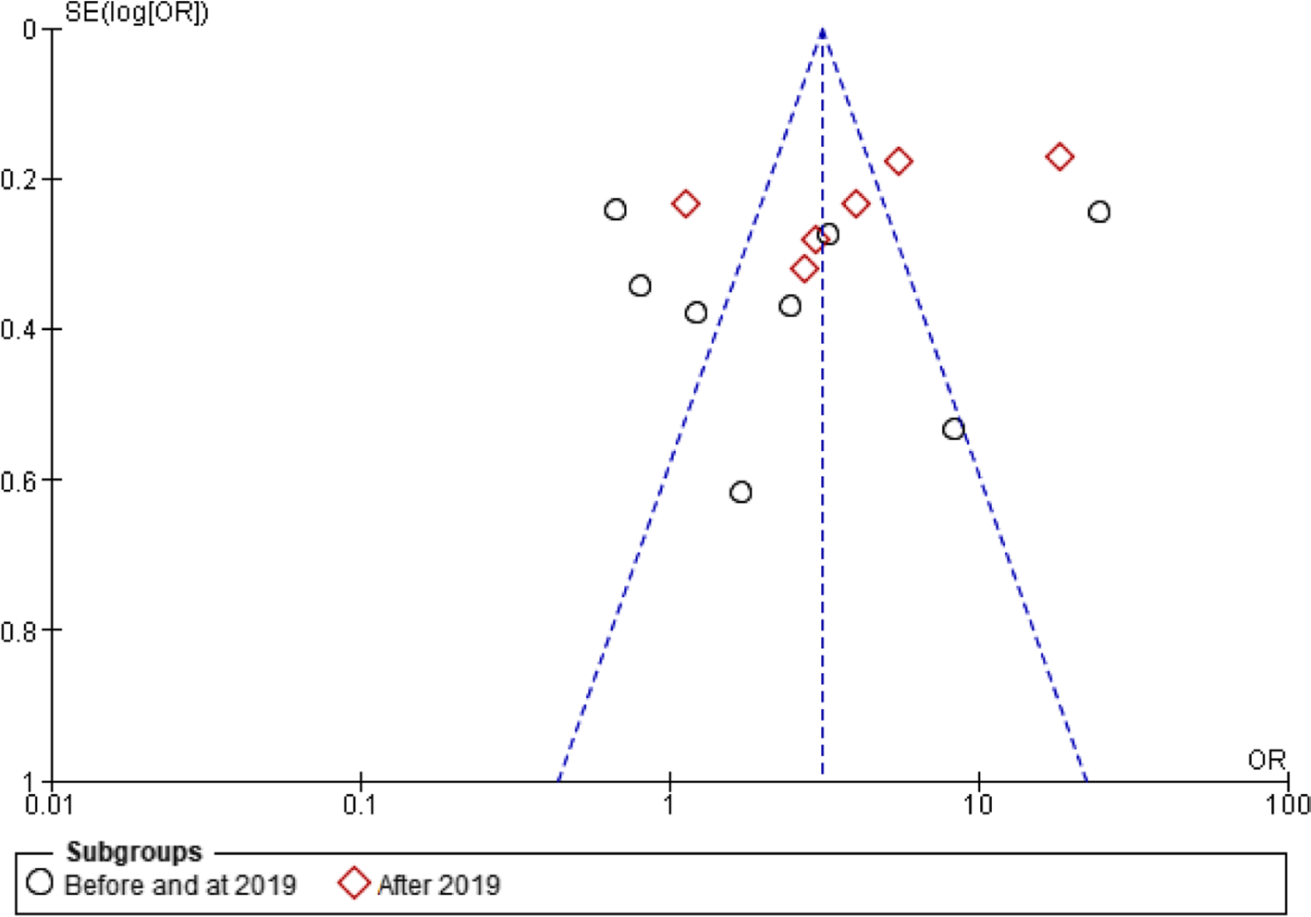
Funnel plot for meta-analysis of the burden of household Out-Of-Pocket healthcare expenditures in Ethiopia, 2021

Failure to include all of the relevant studies that have been conducted in a meta-analysis is attributed to publication bias [58]. In addition, during the analysis of publication bias, larger studies with higher power are placed towards the top and lower-powered studies are placed towards the bottom in the funnel plot [59]. Publication bias was assessed using visual inspection of the funnel plot whether there is asymmetry or not [59]. In this review, the result indicated that studies were placed towards the top; however, the funnel plot illustrates asymmetry due to heterogeneity. Publication of studies depends on the nature and direction of their results and is more likely if the results are significant or perceived as important [58]. In this review, articles that exhibited odds ratio < 1, odds ratio between 1 and 10, and odds ratio > 10 were identified and included in the meta-analysis (Fig. 3). In addition, the figure illustrated that there were missing studies on the bottom left-hand side of the plot. Therefore, since most of this area contains regions of high significance, publication bias is unlikely to be the underlying cause of asymmetry.

## Discussion

In this systemic review and meta-analysis, the purpose was to provide a comprehensive understanding of the burden of financial hardship for healthcare-seeking in Ethiopia and to advance research by generating novel national evidence (pooled estimates) household Out-Of-Pocket healthcare expenditures and their influencing factors with the aim to assess the burden of household Out-Of-Pocket healthcare expenditures in Ethiopia. The review question was: what was the extent of the burden of Out-Of-Pocket healthcare expenditures that were associated with poor socioeconomic status, compared to the rich, in Ethiopian households with illnesses?

This systematic review and meta-analysis included 27 original studies addressing the burden of household Out-Of-Pocket healthcare expenditures in Ethiopia. Regardless of the source and heterogeneity, this study revealed that the pooled burden household Out-Of-Pocket healthcare expenditure in Ethiopia was strongly positively associated with household economic status. The odds of facing Out-Of-Pocket / catastrophic healthcare expenditures among the poorest quintile were about three times that of the richest. In addition, on pooled analysis, the mean direct Out-of-Pocket healthcare expenditures were $32 per month and the mean indirect Out-Of-Pocket healthcare expenditures were $15 per month. About 40% of the households faced disproportionately higher catastrophic health expenditure at 10% of the threshold. Moreover, 40% of the households used a sale of household assets, support from family, or loan as the common coping mechanisms.

For example, this finding suggests that a significant proportion of Ethiopian households or individuals face catastrophic healthcare expenditure which is an obstacle for basic access to healthcare and utilization. This finding is higher than the studies conducted in Rwanda and Tanzania reported that 20.1 and 18% of the households had catastrophic health expenditure respectively [60, 61]. Similarly, the number of households categorized as having catastrophic health expenditure in Kenya was 18% at a 30% threshold to 22.2% at a 10% threshold [62]. Yet, this finding is consistent with the findings from south Ethiopia in which 46% of households faced catastrophic healthcare expenditure at the threshold of 10% of total household expenditure [14]. The possible explanation for the observed difference might be due to socioeconomic differences between the households of Iran and Ethiopia. However, even though Out-Of-Pocket / catastrophic healthcare expenditures are increasing, countries with a prepayment system or social protection system provide better access to healthcare and are less burdened by Out-Of-Pocket / catastrophic healthcare expenditures [63]. Therefore, it should be noted that other potential causes may also exist, such as lack of free healthcare in public health facilities and changes in the incidence of diseases towards more chronic illnesses.

In this review, the common influencing factors on the burden of household Out-Of-Pocket healthcare expenditures in Ethiopia were socioeconomic and demographic factors (i.e., age, household wealth / income, family size, place of residence, educational status, marital status, and occupation); environmental factors (i.e., distance to health facility, and type of health facility visited); illness status (i.e., having chronic illness / any illness); and having health insurance, however, after excluding factors with high heterogeneity, factors such as educational status and having a chronic illness/any illness were not heterogeneous; and therefore, the combined effect size of these two factors was further interpreted here.

According to previous studies, households with lower educational status are at an increased risk of incurring the burden of household Out-Of-Pocket / catastrophic healthcare expenditures [64, 65]. In this review, educational status was associated with the burden of household Out-Of-Pocket / catastrophic healthcare expenditures. This is also evidenced by other studies from Ethiopia indicating that patients with no formal education have a higher total cost of hypertension illness [50] and have higher pre-and post-diagnosis costs of tuberculosis patients compared to patients with a primary educational level [34]. These findings are consistent with a study from China indicating households someone with higher education are less likely to suffer catastrophic health expenditure [66]. Household heads with little education increased the odds of catastrophic health expenditure within the poor [67]. This requires an integrated, poverty-oriented social policy approach is needed to address these factors and alleviate the burden of household Out-Of-Pocket / catastrophic healthcare expenditures.

In this review, concerning the presence of chronic illness / any illness in the household was found to be statistically significant with household Out-Of-Pocket or catastrophic healthcare expenditures. This is evidenced by a similar finding from Malawi in which suffering from a chronic illness has a significant positive association with Out-Of-Pocket / catastrophic healthcare expenditures [68]. In the case of chronic illness, doubling household Out-Of-Pocket healthcare expenditure is consistent with another finding in a study from Bangladesh’s population [69]. Moreover, in Ethiopia, the odds of facing catastrophic healthcare expenditures among hospitalized subjects was about eight times that of the non-hospitalized subjects [44]. Our finding that hospitalizations increased the incidence of catastrophic health expenditures is consistent with findings from other studies from developing countries, Malawi, in which having at least one household member hospitalized increased the odds of catastrophic health expenditures [70]. These effects are common in Low and Middle-Income Countries (LMICs) where many households rely on Out-Of-Pocket for payment of healthcare services. Such reliance places financial burden on households which may leads to catastrophic expenditure and poverty and leaves members of the households in a vicious circle of poverty and ill health. Therefore, the findings of this systematic review and meta-analysis, advance the need to understand the extent of burden of household Out-Of-Pocket healthcare expenditures and its associated risk factors in Low and Middle-Income Countries (LMICs), including Ethiopia, to design and strengthen strategies for financial protection at national level.

### Strength and limitations of the study

To the best of our knowledge, this meta-analysis seems to be the first of its kind in Ethiopia to estimate the pooled estimates of the burden of household Out-Of-Pocket healthcare expenditures in Ethiopia. We also identified the influencing factors of Out-of-Pocket healthcare expenditures / catastrophic healthcare expenditure. However, our study has the following potential limitations both at the study and review level, which may affect the overall conclusions reached. First, for example, at a study level, the included economic studies varied in methodological rigour and quality of reporting, and amount of evidence cited to support claims. The study designs of the studies investigating the burden of household Out-Of-Pocket healthcare expenditures have to be evaluated against the standards expected in current practice. The reliability of different methods of evaluation of household Out-Of-Pocket healthcare expenditures will also have to be considered. Second, despite the drawbacks of our inclusion criteria, a benefit is that our results provide a clearer and more relevant assessment of the economic impact of the burden of household Out-Of-Pocket healthcare expenditures in Ethiopia. At a review level also, as discussed within the practical issues, study heterogeneity in definitions of household Out-Of-Pocket healthcare expenditures outcomes may affect the quality of pooled data. The issue of forgoing treatments was also not captured in the review process.

## Conclusions

This systematic review and meta-analysis revealedthe evidence of inequity in financial hardship that the burden of household Out-Of-Pocket / catastrophic healthcare expenditures gap persists between the richest and the poorest Ethiopian households that is unfair and unjust. Our study also highlights relevant policy variables such as socioeconomic, education, and presence of illness gradient in the burden of household Out-Of-Pocket / catastrophic healthcare expenditures in Ethiopia. Nearly two out of four households experienced catastrophic healthcare expenditure in Ethiopia. The poorest households were more affected by Out-Of-Pocket / catastrophic healthcare expenditures. Being poor, not having a formal education, and having chronic illness were found to be predictors of the burden of household Out-Of-Pocket healthcare expenditures in Ethiopia. The negative impact of chronic illness in terms of increased household health expenditure has been demonstrated. Households resorted to various coping strategies to meet the burden of household Out-Of-Pocket / catastrophic healthcare expenditures on seeking healthcare during illness. Hence, reduction of financing hardship requires reduction of the burden of household Out-Of-Pocket / catastrophic healthcare expenditures.

Therefore, to reduce the detected disparities in seeking healthcare among Ethiopian households, national healthcare priorities should target poor households. This calls for the Ministry of Health to improve the challenges and their impact on equity and design better prepayment policies and strengthen financial protection strategies to protect more vulnerable Ethiopian households.. Furthermore, as inequity in the financial hardship in healthcare-seeking is a major concern in Ethiopia, households facing high Out-Of-Pocket / catastrophic healthcare expenditures might forgo receiving healthcare because of unaffordable charges. Therefore, this issue requires further investigation on-going debates and research in improving the Ethiopian healthcare system.

Finally, based on our study, we suggest examining the feasibility of Community-Based Health Insurance (CNHI) initiatives that pools members’ premium payments into a collective fund. This is because while the government is trying to expand financial protection mechanism, a large segment of the population is still suffering from financial hardship during seeking healthcare, especially the rural poorest households, Besides improving an individual’s education may also be a long-term strategy and can also contribute to a more equitable burden of household Out-Of-Pocket healthcare expenditure and further equitable health.

### Policy implications

This review highlights a base for key policy implications as the burden of household Out-Of-Pocket healthcare expenditure is an indicator of the effectiveness of the current healthcare financing schemes in Ethiopia. Although the burden of household Out-Of-Pocket healthcare expenditure varies depending on the approach and study design, the problem of household Out-Of-Pocket / catastrophic healthcare expenditure in Ethiopia cannot be denied. Therefore, the government of Ethiopia needs to strengthen the current community-based health insurance for rural households and social health insurance for the formal sector. Vulnerable groups, such as low educational status, low-income households, and households with chronic / any illness, should be a priority in the improvement of access to essential healthcare.

## Supplementary Information

**Additional file 1.** Completed PRISMA 2020 checklist. The checklist was used to highlight the important components addressed while preparing this manuscript of systematic review and meta-analysis.

**Additional file 2.** Data extraction summary table or abstraction format. The table was used to present the ways of data collection (study characteristics and outcome measures) in Microsoft excel format. It also contains raw data for outcome analyses.

**Additional file 3.** The Joanna Briggs Institute’s (JBI) standardized critical appraisal tools or checklist used to assess the trustworthiness, relevance, and results of published papers_2017.

**Additional file 4.** A modified version of ‘The Newcastle-Ottawa Scale (NOS)’ quality assessment scale.

## Data Availability

All relevant data are available within the paper and as supplementary files.

## Abbreviations

OOP: Out-Of-Pocket healthcare expenditure
CHE: Catastrophic healthcare expenditures
JBI: Joanna Briggs Institute standardized critical appraisal tools
NOS: The Newcastle-Ottawa Scale quality assessment scale
LMICs: Low and Middle-Income Countries
